# Clinical characteristics and economic burden of hospitalized patients with acute exacerbation of chronic obstructive pulmonary disease in the Chinese Yi population

**DOI:** 10.3389/fmed.2025.1519102

**Published:** 2025-07-31

**Authors:** Ping Li, Meng Li, Weiling Cai, Lian Liu, Dan Xu, Lei Chen, Diandian Li, Mei Chen, Yanqiu Wu, Yongchun Shen, Fuqiang Wen

**Affiliations:** ^1^Department of Pulmonary and Critical Care Medicine, West China Hospital, Sichuan University, Chengdu, China; ^2^State Key Laboratory of Respiratory Health and Multimorbidity, West China Hospital, Sichuan University, Chengdu, China; ^3^Division of Pulmonary Diseases, State Key Laboratory of Biotherapy of China, West China Hospital, Sichuan University, Chengdu, China; ^4^Department of Pulmonary and Critical Care Medicine, Liangshan Hospital of Integrated Traditional and Western Medicine, Xichang, China; ^5^Department of Respiratory and Critical Care Medicine, Luojiang District People’s Hospital, Deyang, China; ^6^Department of High Altitude Medicine, Center for High Altitude Medicine, West China Hospital, Sichuan University, Chengdu, Sichuan, China; ^7^School of Medical and Life Sciences, Chengdu University of Traditional Chinese Medicine (TCM) and Key Laboratory of Acupuncture for Senile Disease, Ministry of Education, Chengdu, China

**Keywords:** acute exacerbation of chronic obstructive pulmonary disease, AECOPD, Chinese Yi population, clinical characteristics, treatment pattern

## Abstract

**Purpose:**

The study aimed to compare the clinical characteristics and economic burden of Yi and Han hospitalized patients with acute exacerbation of chronic obstructive pulmonary disease (AECOPD).

**Patients and methods:**

The patients hospitalized for AECOPD were retrospectively enrolled at the Hospital of Integrated Traditional and Western Medicine in Liangshan Yi Autonomous Prefecture, China, from January 2020 to October 2022. Data regarding the characteristics, treatment, clinical outcomes, and economic burden of Yi and Han AECOPD patients included in the cohort were compared and analyzed. Propensity score matching (PSM) adjusted for age and gender differences was used to assess differences between the two groups.

**Results:**

Among 685 patients (297 Yi, 388 Han), smoking prevalence was similar (Yi 57.6% vs. Han 55.9%, *P* > 0.05). Yi patients were significantly younger (66.3 ± 11.0 vs. 76.2 ± 9.9 years, *P* < 0.001) with more females (36.4% vs. 27.8%, *P* = 0.017). After PSM, Yi patients showed: (1) lower comorbidity rates (coronary heart disease, hypertension, diabetes, and atrial fibrillation); (2) distinct laboratory profiles (higher hemoglobin, platelets, low-density lipoprotein cholesterol; lower D-dimer, PaO_2_, and albumin); (3) reduced intensive care unit (ICU) transfers (*P* < 0.05) and non-invasive ventilation use (*P* < 0.05); and (4) more frequent pulmonary infections (45.1% vs. 35.8%, *P* = 0.014) in unmatched analysis.

**Conclusion:**

Significant ethnic differences exist in AECOPD presentation and management between Yi and Han populations, independent of age and gender. These findings highlight the need for ethnicity-tailored COPD management strategies in multi-ethnic regions.

## Introduction

Chronic obstructive pulmonary disease (COPD) is a prevalent chronic respiratory disorder characterized by persistent airflow limitation and represents a major global health challenge. As the fourth leading cause of death worldwide, COPD accounted for approximately 5% of global mortality in 2021 (1), imposing a substantial burden on both individuals and healthcare systems (2, 3). In China, the disease is particularly pervasive, with a prevalence of 13.7% among adults aged 40 and older (4), establishing COPD as the most common respiratory condition and a major contributor to the national disease burden (5). This burden is projected to escalate due to population aging and worsening environmental pollution (6–9).

Acute exacerbations of COPD (AECOPD)—defined as acute worsening of respiratory symptoms (e.g., cough, sputum production, and dyspnea) (10) necessitating intensified treatment—accelerate lung function decline and disease progression while placing strain on healthcare systems (11–14). Annually, 22%–40% of COPD patients experience at least one moderate-to-severe exacerbation, with 9%–16% suffering recurrent episodes (12, 15). Notably, AECOPD constitutes the largest share of COPD-related healthcare expenditures (16, 17) and is strongly linked to increased mortality risk (18).

Geographical and ethnic disparities further complicate COPD epidemiology in China. Striking regional variations exist, with prevalence ranging from 20.2% in the southwest to 10.2% in central regions (19), while ethnic minority populations exhibit disproportionately higher rates (20). This pattern likely reflects interethnic differences in both behavioral risk factors (particularly tobacco use) and environmental exposures, including household air pollution from biomass fuels and occupational hazards (20, 21). The observed heterogeneity extends beyond incidence to encompass three critical clinical dimensions: disease manifestation and progression patterns (22, 23); comorbidity profiles and mortality rates (24); and therapeutic responses to pharmacological and non-pharmacological interventions (25, 26). This multifaceted variation underscores the complex interplay of genetic predisposition, cultural practices, and environmental influences in COPD pathogenesis. Notably, while global research has established these ethnic disparities, critical gaps persist in understanding their implications for AECOPD. Most conspicuously, comparative studies between China’s dominant Han population and ethnic minority groups (e.g., Yi) remain lacking—a significant omission given China’s unique multi-ethnic context and the clinical importance of AECOPD in disease prognosis. This knowledge gap substantially limits our ability to develop ethnically tailored management strategies for acute exacerbations.

This study focuses on the Yi ethnic group, one of China’s 55 officially recognized minorities, residing predominantly in the mountainous Liangshan Yi Autonomous Prefecture of southern Sichuan Province (27). As a culturally distinct population with documented health disparities, the Yi community remains understudied in respiratory research despite exhibiting distinct socioeconomic determinants: the Yi community relies primarily on biomass fuels for cooking (28) and has significantly higher smoking rates than Han populations (29). This research gap is especially concerning given Liangshan’s status as a COPD hyperendemic region. To address this, we conducted a comparative analysis of clinical characteristics, treatment outcomes, and economic burdens between Yi and Han Chinese patients hospitalized for AECOPD, providing the first systematic comparison of interethnic differences in acute exacerbation management in this high-risk population.

## Patients and methods

### Study design and participants

We conducted a retrospective cohort study of 685 AECOPD patients who underwent chest CT scans at the Department of Respiratory and Critical Care Medicine of Liangshan Yi Autonomous Prefecture Hospital of Integrated Traditional Chinese and Western Medicine between January 2020 to October 2022. Patients were identified using ICD-10 codes for COPD from the hospital’s electronic medical records system, with ethnicity confirmed via national ID card registration. The inclusion criterion was hospitalized AECOPD patients with ≥1 acute worsening clinical symptom, including cough, sputum production, and/or dyspnea, with a hospital stay of >2 days. This study was approved by the ethics committee of the Hospital of Integrated Traditional and Western Medicine in Liangshan Yi Autonomous Prefecture, and written informed consent was waived due to the retrospective nature (approval number: 2023-8-7). The exclusion criteria are as follows: (1) the hospital stay was less than 2 days; (2) combined with other respiratory diseases, such as bronchiectasis, asthma, tuberculosis, pulmonary fibrosis, lung cancer, etc.; (3) combined with severe organ dysfunction, such as cardiopulmonary resuscitation on admission, acute myocardial infarction, cerebrovascular accident, renal failure need dialysis, etc.; and (4) had no major clinical data and laboratory data; for patients who had multiple AECOPD admissions during the study period, only the first admission was included.

### Data collection

Collected data included demographic characteristics, COPD exacerbations in the previous year, comorbidities, symptoms and signs on admission, laboratory (including blood routine examination, liver and kidney function, arterial blood gas, coagulation function, etc.), and radiologic findings. Smoking exposure was systematically assessed through comprehensive patient interviews, retrospective review of medical records, and calculation of pack-year history using standardized nursing admission questionnaires. For analytical purposes, smokers were operationally defined as individuals with a cumulative tobacco exposure of ≥10 pack-years, encompassing both current and former smokers. The radiologic images were interpreted by the radiologist, who was informed that pneumonia was clinically suspected but had no other medical information and was not directly involved in the study. The clinician then combined the CT results with the radiologist’s interpretation to evaluate the probability of a pulmonary infection.

### Clinical outcomes

In terms of clinical outcomes, we evaluated outpatient medication, in-hospital mortality, non-invasive ventilator utilization rate, ICU admissions, length of stay (LOS), and total hospitalization costs.

### Statistical analysis

Use Excel to collect and organize data. For continuous data, an independent sample *t*-test was used for analysis, and the results were expressed as “mean ± standard deviation.” The categorical data were tested by the Chi-square test. Patients with incomplete data in any of the study variables or who were lost to follow-up were excluded from the final analytical cohort. To address ethnic disparities in AECOPD manifestations, propensity score matching (PSM) was implemented to adjust for potential confounding between Yi and Han populations. Key matching covariates included core demographic parameters (age and sex) and modifiable risk factors (smoking status). *P* < 0.05 was considered statistically significant. All analyses were performed using SPSS version 25.0.

## Results

### Baseline clinical characteristics of study participants

**FIGURE 1:**
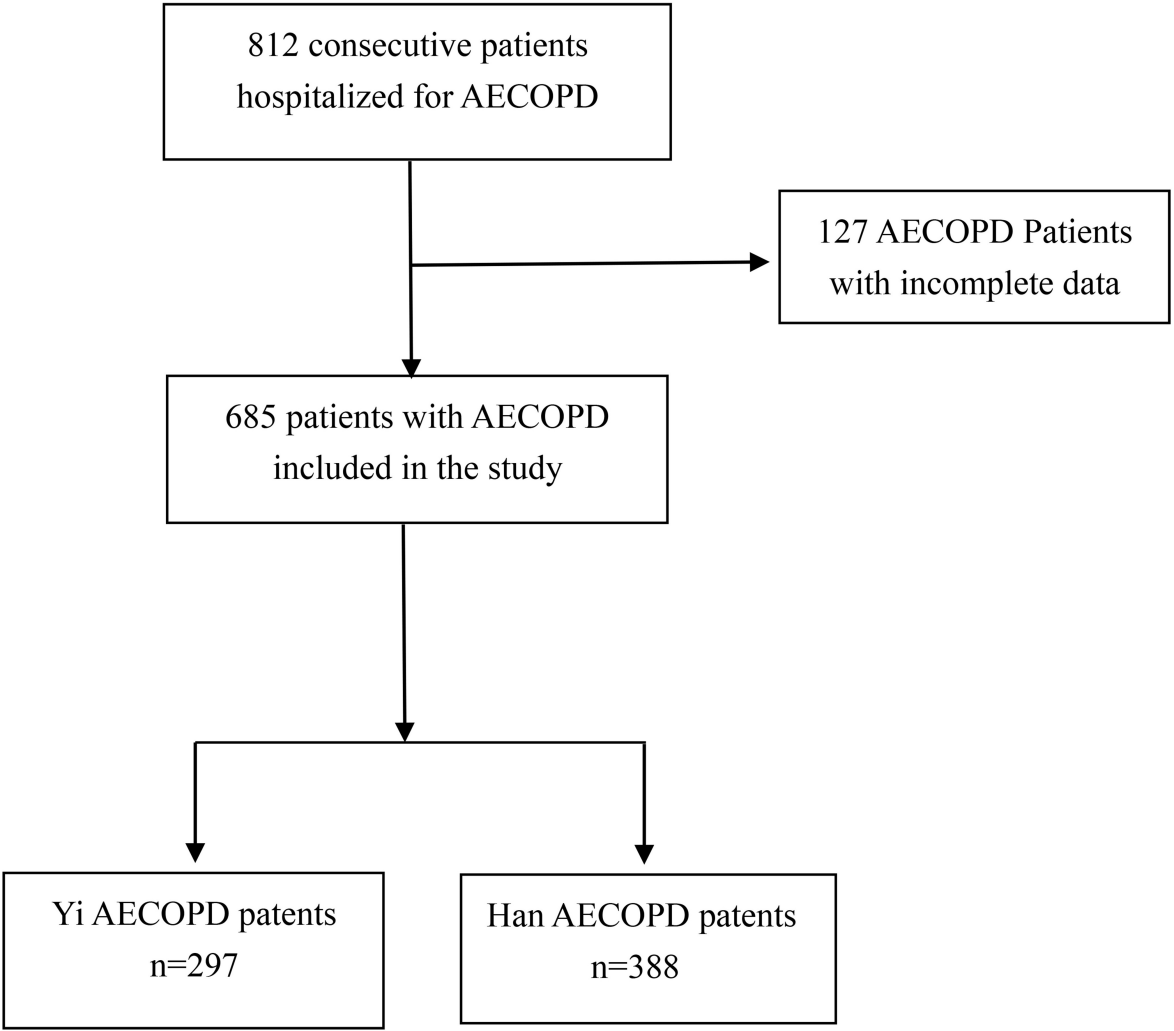
Flow chart of the study.

In total, 685 eligible AECOPD patients with a rate of 43.4% of non-smokers were included in this study, which was categorized into two groups: Yi (*n* = 297) and Han (*n* = 388) groups (Figure 1). Before PSM, the characteristics of Yi and Han AECOPD patients at baseline were significantly different in terms of age (66.25 ± 11.02 vs. 76.16 ± 9.93, *P* < 0.001) and gender (male) (63.6% vs. 72.2%, *P* = 0.017). Table 1 describes and compares the characteristics of patients before and after PSM. The smoking rate among Yi patients was higher than that of Han patients before and after PSM, but the difference was not statistically significant (*P* > 0.05). The three acute exacerbations in the previous year were significantly different before and after PSM. In terms of outpatient medication treatments, the proportion of Han patients receiving drug treatment was higher than that of Yi patients (*P* < 0.05).

**TABLE 1:** Baseline characteristics and comorbidities of the study patients.

Variable	Before PSM				After PSM			
	Total (*N* = 685)	Yi ethnic (*N* = 297)	Han ethnic (*N* = 388)	*P*-value	Total (*N* = 412)	Yi ethnic (*N* = 206)	Han ethnic (*N* = 206)	*P*-value
Gender, male (*n*, %)	469 (68.5)	189 (63.6)	280 (72.2)	0.017	267 (64.8)	135 (65.5)	132 (64.1)	0.757
Age, years	71.86 ± 11.51	66.25 ± 11.02	76.16 ± 9.93	<0.001	70.56 ± 9.10	70.44 ± 8.87	70.67 ± 9.34	0.218
Smoker (*n*, %)	388 (56.6)	171 (57.6)	217 (55.9)	0.248	244 (59.2)	129 (62.6)	115 (55.8)	0.160
Current smoker (*n*, %)	166 (24.2)	81 (27.3)	85 (21.9)		113 (27.4)	59 (28.6)	54 (26.2)	
Former smoker (*n*, %)	222 (32.4)	90 (30.3)	132 (34.0)		131 (31.8)	70 (34.0)	61 (29.6)	
**Frequency of hospitalization due to AECOPD in the past year (times) (n, %)**								
1	562 (82.0)	246 (82.8)	316 (81.4)	0.64	335 (81.3)	170 (82.5)	165 (80.1)	0.527
2	101 (14.7)	46 (15.5)	55 (14.2)	0.631	63 (15.3)	33 (16.0)	30 (14.6)	0.681
3	22 (3.2)	5 (1.7)	17 (4.4)	0.047	14 (3.4)	3 (1.5)	11 (5.3)	0.028
Outpatient medication treatments (*n*, %)	68 (9.9)	19 (6.4)	49 (12.6)	0.005	39 (9.5)	11 (5.3)	28 (13.6)	0.004
Inhaled short-acting bronchodilator (*n*, %)	9 (1.3)	4 (1.3)	5 (1.3)		7 (1.7)	4 (1.9)	3 (1.5)	
Oral glucocorticoids (*n*, %)	10 (1.5)	4 (1.3)	6 (1.5)		6 (1.5)	1 (0.4)	5 (2.4)	
Inhaled ICS/LABA (*n*, %)	48 (7.0)	11 (3.7)	37 (9.5)		25 (6.1)	6 (2.9)	19 (9.2)	
Traditional Chinese medicine (*n*, %)	1 (0.1)	0 (0)	1 (0.3)		1 (0.2)	0 (0)	1 (0.4)	

**FIGURE 2:**
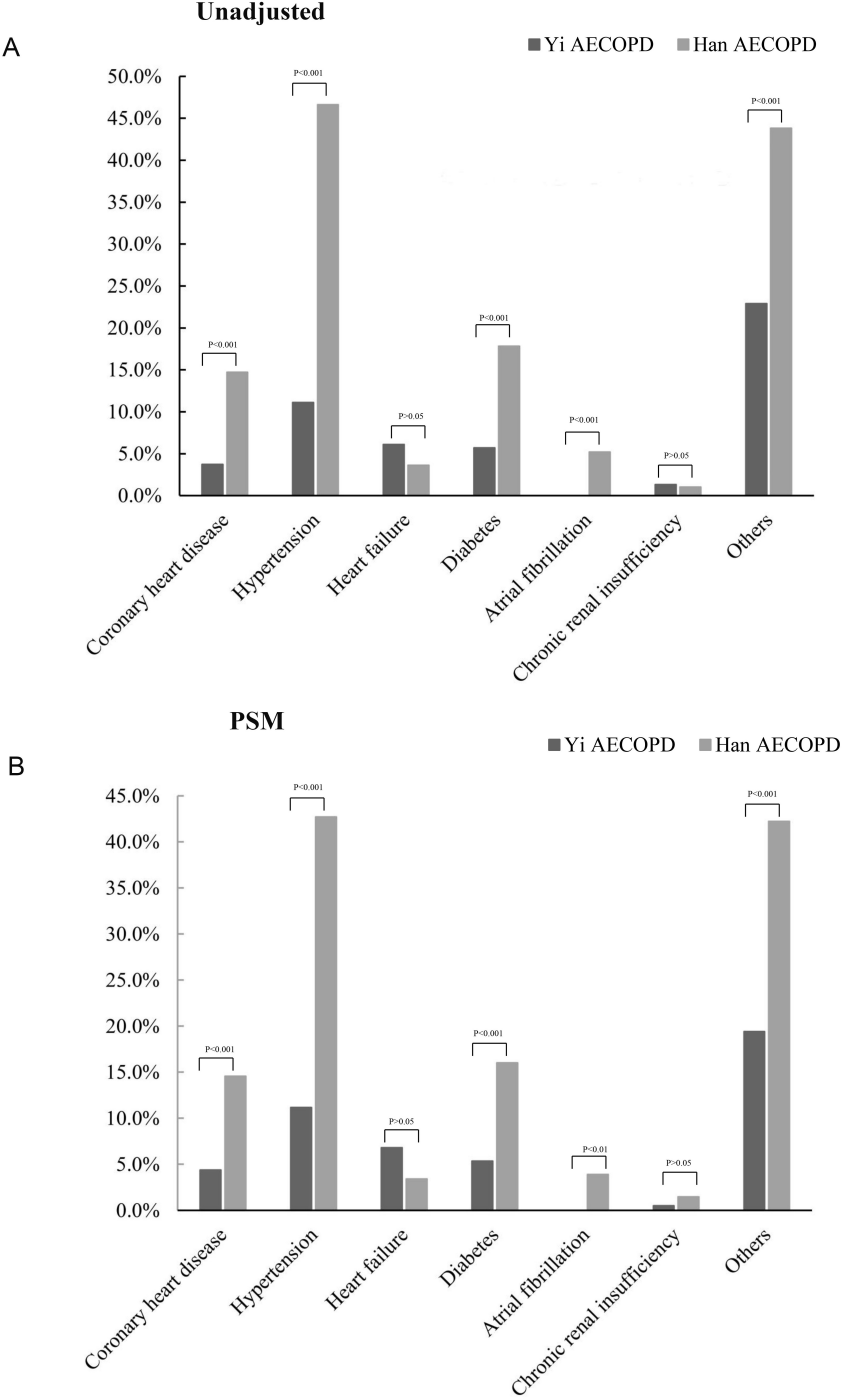
The incidence of comorbidities in AECOPD of Yi and Han. **(A)** The incidence of comorbidities was not matched for analysis; **(B)** the incidence of comorbidities after propensity matching analysis.

The most common complications were hypertension and diabetes in the overall cohort. Both unadjusted and PSM-adjusted analyses revealed Yi patients had significantly lower rates of coronary heart disease, hypertension, diabetes, and atrial fibrillation compared to Han counterparts (Figure 2).

### Clinical symptoms and signs

Our study showed that before matching, Han patients had a higher incidence of dyspnea/breathing difficulty (86.9% vs. 80.8%, *P* = 0.031), while Yi ethnic patients had a higher rate of fever (11.8% vs. 6.7, *P* = 0.021) and wheezing (70.0% vs. 62.1%, *P* = 0.031) in the unadjusted. However, there was no difference in symptoms between the two groups after matching (Table 2). Regarding vital signs on admission, Yi ethnic patients tended to have lower systolic blood pressure (SBP), pulse rate, respiratory rate, and oxygen saturation concentration (all *P* < 0.05) before and after PSM. There was no significant difference in diastolic blood pressure (DBP) between the two groups before unadjusted, but after matching (Table 2).

**TABLE 2:** Clinical features of the study patients.

Variable	Before PSM				After PSM			
	Total (*N* = 685)	Yi ethnic (*N* = 297)	Han ethnic (*N* = 388)	*P*-value	Total (*N* = 412)	Yi ethnic (*N* = 206)	Han ethnic (*N* = 206)	*P*-value
**Symptoms (n, %)**								
Cough	577 (84.2)	294 (99.0)	382 (98.5)	0.541	344 (83.5)	166 (80.6)	178 (86.4)	0.111
Expectoration	676 (98.7)	294 (99.0)	378 (97.4)	0.136	410 (99.5)	205 (99.5)	205 (99.5)	1
Dyspnea/breathing difficulty	672 (98.1)	240 (80.8)	337 (86.9)	0.031	407 (98.8)	205 (99.5)	202 (98.1)	0.177
Fever	61 (8.9)	35 (11.8)	26 (6.7)	0.021	36 (8.7)	23 (11.1)	13 (6.3)	0.081
Wheeze	449 (65.5)	208 (70.0)	241 (62.1)	0.031	281 (68.2)	147 (71.4)	134 (65.0)	0.169
**Signs**								
SBP (mmHg)	123.41 ± 21.83	117.51 ± 20.96	127.93 ± 21.43	<0.001	122.75 ± 21.58	118.09 ± 21.25	119.09 ± 21.25	<0.001
DBP (mmHg)	74.28 ± 12.51	73.32 ± 12.56	75.02 ± 12.44	0.078	74.65 ± 12.81	73.03 ± 12.89	76.26 ± 12.56	0.01
Pulse rate (bpm)	86.67 ± 19.26	84.15 ± 18.69	88.6 ± 19.49	0.003	86.70 ± 18.89	83.85 ± 17.79	89.55 ± 18.61	0.02
Respiratory rate (bpm)	20.08 ± 1.68	19.85 ± 1.19	20.27 ± 1.96	0.001	20.05 ± 1.59	19.89 ± 1.35	20.21 ± 1.79	0.044

SBP, systolic blood pressure; DBP, diastolic blood pressure.

### Laboratory and radiographic findings

The laboratory and radiographic findings of the study patients are summarized in Table 3. Before matching, compared to Han patients, Yi patients had higher levels of hemoglobin, platelet, arterial blood gas potential of hydrogen, and high-density lipoprotein cholesterol (HDL-C) and had lower levels of D-dimer, partial pressure of oxygen, albumin, creatinine, uric acid levels, glucose, low-density lipoprotein cholesterol (LDL-C). There were no significant differences between the Yi ethnic and Han groups in terms of white blood cells (absolute neutrophil count, absolute lymphocyte count, absolute eosinophil count, and eosinophil percentage), electrolytes, prothrombin time, activated partial prothrombin time, partial carbon dioxide pressure, bicarbonate ion, blood oxygen saturation, C-reactive protein (CRP), procalcitonin (PCT), brain natriuretic peptide, and electrolyte. In CT findings, pleural effusion and pulmonary infection were the most common in the overall cohort, while pulmonary infection was more common in Yi ethnic patients (45.1% vs. 35.8%, *P* = 0.014). However, after matching, there were no significant differences between Yi and Han in hemoglobin, uric acid levels, LDL-C, D-dimer, PaO_2_, and pulmonary infection, and significant differences in PCO_2_ and K^+^ (Table 3).

**TABLE 3:** Laboratory and CT findings of this study.

Variable	Before PSM				After PSM			
	Total (*N* = 685)	Yi ethnic (*N* = 297)	Han ethnic (*N* = 388)	P-value	Total (*N* = 412)	Yi ethnic (*N* = 206)	Han ethnic (*N* = 206)	P-value
**Laboratory tests**								
**Blood routine findings**								
Hemoglobin (g/L)	141.05 ± 24.24	144.86 ± 24.61	138.14 ± 23.58	<0.001	142.28 ± 23.69	143.06 ± 24.48	141.00 ± 22.90	0.504
Platelet (×10^9^/L)	211.34 ± 88.04	235.54 ± 89.46	192.83 ± 82.38	<0.001	212.77 ± 88.59	234.16 ± 92.57	193.39 ± 79.55	<0.001
WBC (×10^9^/L)	8.15 ± 3.6	8.38 ± 3.56	7.98 ± 3.63	0.149	7.94 ± 3.22	8.24 ± 3.37	7.62 ± 3.04	0.051
NEUT (×10^9^/L)	6.39 ± 4.35	6.37 ± 3.54	6.41 ± 4.89	0.892	6.11 ± 3.13	6.33 ± 3.35	5.88 ± 2.89	0.145
Lymphocyte (×10^9^/L)	1.51 ± 4.87	1.46 ± 0.63	1.56 ± 6.45	0.785	1.59 ± 6.19	1.34 ± 0.60	1.84 ± 8.74	0.415
Eosinophil (×10^9^/L)	0.13 ± 0.34	0.11 ± 0.15	0.13 ± 0.44	0.473	0.11 ± 0.117	0.11 ± 0.15	0.12 ± 0.18	0.504
EOSR (%)	1.65 ± 2.21	1.66 ± 2.21	1.64 ± 2.22	0.93	1.61 ± 2.09	1.60 ± 2.23	1.61 ± 1.95	0.961
**Liver function findings**								
AST	27.27 ± 47.47	25.77 ± 17.53	28.41 ± 61.02	0.473	27.14 ± 49.77	27.78 ± 16.14	24.48 ± 68.35	0.34
ALT	24.07 ± 36.64	23.80 ± 25.25	24.27 ± 43.16	0.869	46.56 ± 73.72	21.19 ± 116.75	25.50 ± 50.58	0.257
Albumin (g/L)	37.84 ± 5.57	37.34 ± 5.14	38.21 ± 5.85	0.044	23.392 ± 38.03	36.87 ± 5.22	38.76 ± 6.27	0.001
**Kidney function findings**								
Creatinine (μmol/L)	74.73 ± 33.14	65.14 ± 18.11	82.01 ± 39.52	<0.001	71.70 ± 28.56	67.37 ± 19.52	76.05 ± 34.87	0.002
Uric acid (μmol/L)	349.18 ± 123.63	332.18 ± 110.53	362.08 ± 131.39	0.001	344.72 ± 121.92	336.10 ± 110.61	353.34 ± 131.98	0.153
**Glucose findings**								
Glucose (mmol/L)	6.80 ± 2.65	6.43 ± 2.35	7.08 ± 2.82	0.001	6.68 ± 2.63	6.40 ± 2.19	6.94 ± 2.96	0.039
**Blood lipid findings**								
HDL-C (mmol/L)	1.27 ± 0.36	1.17 ± 0.29	1.32 ± 0.38	<0.001	1.39 ± 0.36	1.20 ± 0.29	1.37 ± 0.39	<0.001
LDL-C (mmol/L)	2.94 ± 1.04	3.14 ± 1.07	2.83 ± 1.01	0.011	3.00 ± 1.05	3.09 ± 1.08	2.93 ± 1.33	0.275
**Electrolyte findings**								
Ca^+^ (mmol/L)	2.49 ± 4.04	2.34 ± 0.13	2.61 ± 5.36	0.388	2.60 ± 5.21	2.32 ± 0.13	2.88 ± 7.35	0.284
Na^+^ (mmol/L)	142.05 ± 46.36	140.17 ± 4.15	143.47 ± 61.35	0.358	140.88 ± 14.09	140.14 ± 3.58	141.62 ± 19.55	0.288
K^+^ (mmol/L)	4.06 ± 0.54	4.04 ± 0.52	4.09 ± 0.54	0.223	4.10 ± 0.54	4.04 ± 0.53	4.15 ± 0.54	0.046
Cl^–^ (mmol/L)	102.26 ± 7.23	102.51 ± 6.87	102.07 ± 7.49	0.429	102.14 ± 8.25	102.30 ± 7.75	101.98 ± 8.73	0.685
**CRP findings**								
CRP (mg/L)	27.24 ± 41.48	28.13 ± 44.18	26.56 ± 39.33	0.626	25.77 ± 40.99	29.16 ± 43.38	38.26 ± 2.68	0.095
**PCT findings**								
PCT (ng/ml)	0.51 ± 4.78	0.7 ± 6.82	0.35 ± 1.15	0.438	0.25 ± 0.48	0.24 ± 0.46	0.27 ± 0.52	0.613
Cardiac function test findings								
BNP (pg/ml)	1,900.69 ± 3,263.29	1,580.4 ± 2,928.8	2,113.53 ± 3,455.89	0.058	1,925.25 ± 3,296.54	1,758 ± 3,144.37	2,079.09 ± 3,433.97	0.395
**D-dimer findings**								
D-dimer (ng/ml)	713.72 ± 971.89	831.83 ± 60.51	1,051.7 ± 65.86	0.004	657.95 ± 858.38	707.27 ± 973.06	611.46 ± 740.53	0.379
Coagulation time findings								
PT (s)	14.87 ± 4.82	14.71 ± 2.48	15.01 ± 6.14	0.452	14.65 ± 3.31	14.76 ± 2.64	14.54 ± 3.92	0.561
APTT (s)	31.37 ± 6.88	30.99 ± 6.31	31.69 ± 7.32	0.246	31.14 ± 6.51	31.13 ± 6.90	31.14 ± 6.08	0.991
**Arterial blood gas findings**								
PH	7.41 ± 0.07	7.42 ± 0.05	7.4 ± 0.08	0.01	7.41 ± 0.07	7.42 ± 0.06	7.40 ± 0.07	0.005
PaO_2_ (mmHg)	64.08 ± 23.73	60.93 ± 19.41	66.11 ± 25.97	0.027	63.07 ± 23.85	61.93 ± 20.13	64.10 ± 26.79	0.508
PaCO_2_ (mmHg)	41.73 ± 11.75	40.85 ± 7.29	42.3 ± 13.87	0.182	42.36 ± 12.92	39.71 ± 6.67	44.73 ± 16.31	0.003
HCO_3_ (mmol/L)	26.14 ± 5.22	26.55 ± 4.03	25.87 ± 5.85	0.18	26.48 ± 5.43	26.11 ± 3.91	26.81 ± 6.50	0.346
SPO_2_ (%)	89.18 ± 7.25	89.08 ± 7	89.26 ± 7.45	0.754	88.80 ± 7,86	89.21 ± 6,73	88.39 ± 8.01	0.274
**CT findings (%)**								
Pleural effusion	119 (17.3)	47 (15.8)	72 (18.6)	0.350	66 (16.0)	35 (17.0)	31 (15.0)	0.591
Pulmonary infection	273 (39.6)	134 (45.1)	139 (35.8)	0.014	45 (10.9)	27 (13.1)	18 (8.7)	0.155

WBC, white blood cell; NEUT, neutrophil ratio; EOSR, eosinophil ratio; BNP, B-type natriuretic peptide; PCT, procalcitonin; CRP, C-reactive protein; PT, prothrombin time; APTT, activated partial thromboplastin time; AST, aspartate aminotransferase; ALT, alanine aminotransferase; CT, computed tomography.

### Clinical outcomes

**FIGURE 3:**
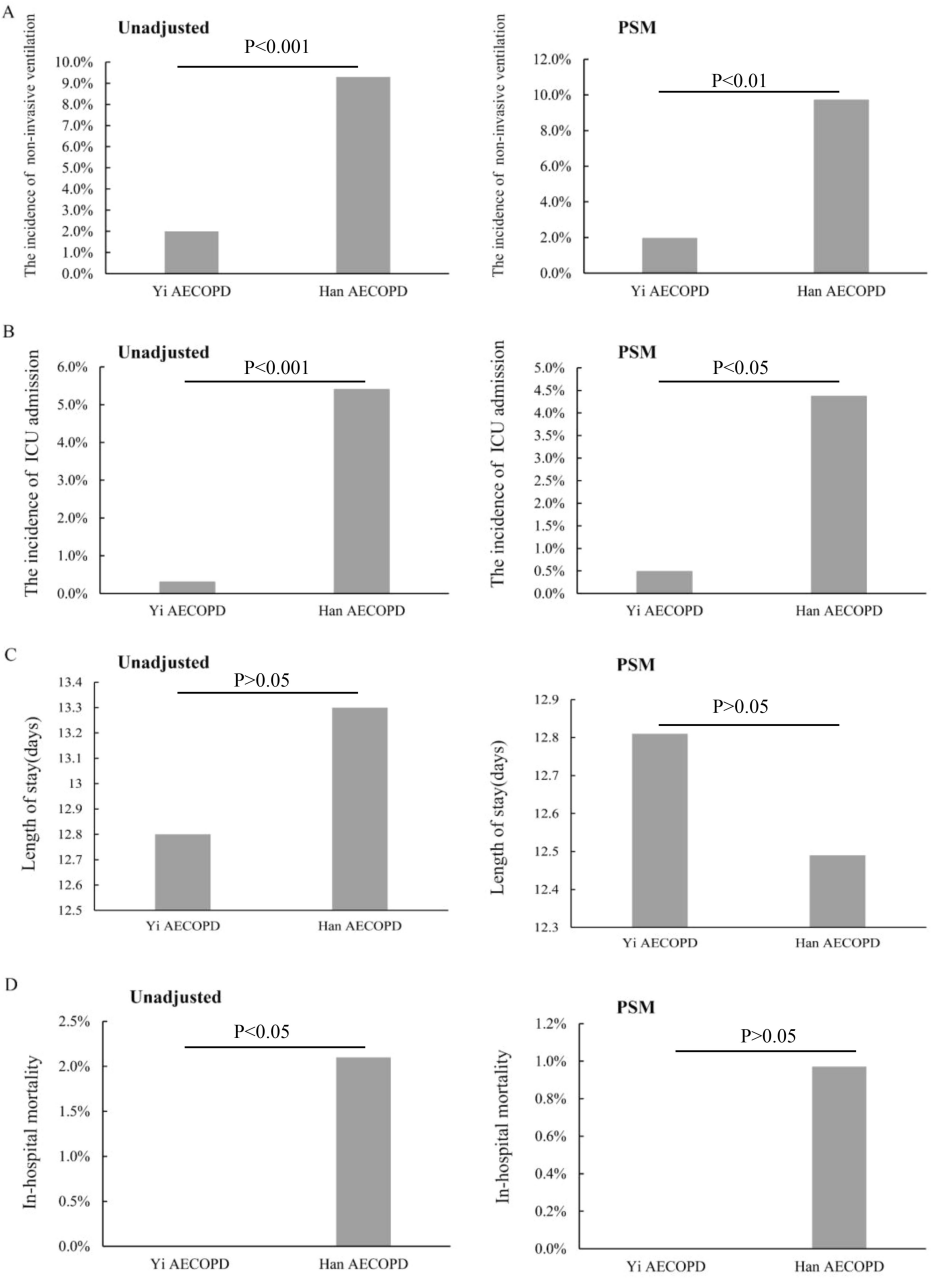
The treatment outcomes. **(A)** The incidence of using non-invasive ventilators; **(B)** the incidence of ICU admission at discharge; **(C)** the length of stay (days); **(D)** the in-hospital mortality.

Both before and after matching, Yi patients with AECOPD were less likely to receive non-invasive ventilators during hospitalization (Figure 3A). The ICU admission of Yi patients was less needed during hospitalization than in Han patients (Figure 3B). However, the mean LOS showed no significant differences between the two groups (Figure 3C). It is worth noting that before matching, the in-hospital mortality of Yi patients was lower than that of Han patients (*P* < 0.05) (Figure 3D), and there was no significant difference between the two groups after matching (*P* > 0.05) (Figure 3D).

### Economic burden

Before matching, our analysis demonstrated significantly lower total hospitalization costs for Yi patients compared to Han patients (12,677.36 ± 7,642.23 CNY vs. 15,195.86 ± 20,652.94 CNY, *P* < 0.05), particularly in examination and treatment fees (Table 4). However, after PSM for key clinical variables, no significant differences in hospitalization costs persisted between Yi and Han patients (*P* > 0.05) (Table 4). This suggests that the initial observed cost disparities were likely mediated by confounding factors rather than ethnicity itself.

**TABLE 4:** The economic burden of this study.

Variable	Before PSM				After PSM			
	Total (*N* = 685)	Yi ethnic (*N* = 297)	Han ethnic (*N* = 388)	*P*-value	Total (*N* = 685)	Yi ethnic (*N* = 297)	Han ethnic (*N* = 388)	*P*-value
Hospitalization cost (CNY)	14,103.9 ± 16,375.84	12,677.36 ± 7,642.23	15,195.86 ± 20,652.94	0.027	13,171.47 ± 11,942.20	12,814.92 ± 8,772.97	13,528.12 ± 14,446.71	0.545
Pharmaceutical treatment intervention costs (CNY)	5,535.92 ± 5,653.92	5,166.34 ± 3,364.08	5,818.82 ± 6,903.51	0.104	5,316.53 ± 4,810.48	3,825.71 ± 266.55	5,635.34 ± 392.63	0.922
Examination costs (CNY)	2,975.66 ± 2,001.78	2,761.69 ± 1,249.59	3,139.44 ± 2,413.72	0.008	2,752.00 ± 1,479.83	2,690.27 ± 1,287.08	2,813.73 ± 1,651.12	0.398
Non-pharmaceutical treatment intervention costs (CNY)	3,341.15 ± 5,473.68	2,776.23 ± 2,318.82	3,773.57 ± 6,957.71	0.009	3,029.59 ± 3,825.38	2,770.55 ± 2,667.53	3,288.64 ± 4,699.77	0.170
Other expenses (CNY)	2,251.18 ± 3,849.52	1,973.1 ± 1,565.96	2,464.04 ± 4,920.47	0.065	2,073.35 ± 2,487.05	2,014.40 ± 1,799.57	2,132.30 ± 3,025.82	0.631

CNY, Chinese yuan.

## Discussion

Acute exacerbation of chronic obstructive pulmonary disease presents with distinct clinical profiles characterized by severe respiratory manifestations, poor prognoses, and substantial healthcare costs (30). While existing literature has extensively documented the clinical characteristics and management of AECOPD (31–33), critical gaps remain in understanding ethnodemographic variations, particularly between China’s Yi and Han populations. Our study enrolled 685 AECOPD patients (297 Yi vs.388 Han) and revealed critical interethnic variations. The smoking rate of Han patients was lower than that of Yi patients before and after matching, but the difference was not statistically significant (*P* > 0.05). Cigarette smoke is widely recognized as a major environmental risk factor for COPD, 43.4% of our cohort were never-smokers, consistent with global reports that 25%–45% of COPD cases occur in non-smokers (34). This substantial burden among non-smokers highlights the importance of alternative risk factors, particularly in the Liangshan context where biomass fuel exposure is ubiquitous. Multiple studies have established biomass combustion as a major risk factor for COPD, especially among non-smoking women (35, 36), and as a significant contributor to respiratory hospitalization risk (37). The demographic composition of our Yi cohort (higher proportion of female patients) combined with regional solid fuel use patterns suggests biomass exposure may be a predominant etiology for non-smoking COPD in this population. These findings underscore the need for targeted interventions to reduce household air pollution in this region. Therefore, reducing or eliminating the use of solid fuel for cooking and heating may be a way to reduce the risk of AECOPD in Yi, which needs to be further confirmed by future studies. The low rate of pre-admission COPD treatment (only 68/685 patients, 9.9%) raises important questions about whether the enrolled patients truly experienced AECOPD or rather manifested untreated, progressive disease. While the study used standard clinical criteria for AECOPD diagnosis, the predominance of treatment-naive patients suggests alternative interpretations: many cases may represent first presentations of advanced COPD rather than true exacerbations, particularly given the high prevalence of risk factors like smoking and biomass exposure in this population. This diagnostic uncertainty could potentially confound the observed ethnic differences, as some variations in clinical presentation and outcomes may reflect disparities in healthcare access and disease management rather than inherent biological differences between Yi and Han populations. The findings highlight both the critical need for improved COPD diagnosis and chronic care infrastructure in this region and the importance of considering treatment status when interpreting exacerbation studies in underserved populations. Future research should incorporate spirometry confirmation and detailed treatment histories to better distinguish true exacerbations from untreated disease progression.

Symptoms of COPD may include chronic and progressive dyspnea, chronic cough, expectoration, chest tightness, or fatigue (3). AECOPD is a serious event in the course of COPD that triggers a catastrophic cascade of reactions that can be overwhelming and life-threatening (18). Previous studies have found that the occurrence of COPD is associated with frequent childhood cough and family history of respiratory disease, suggesting that recurrent pulmonary infections play an important role in COPD (38, 39). Respiratory infection is one of the environmental risk factors of COPD, plays an important role in the onset and progression of COPD, and may be a risk factor for COPD (40, 41). Initial unadjusted analyses suggested higher rates of fever and CT-evident pulmonary infections in Yi AECOPD patients compared to Han patients, potentially indicating greater pneumonia prevalence. However, after PSM analysis, we found no significant difference in the incidence of pulmonary infection between Yi and Han AECOPD patients (*P* > 0.05). This result indicates that although unmatched analysis showed higher rates of fever and CT evidence of pulmonary infection in Yi patients, these differences disappeared after balancing confounding factors such as age and smoking status. The disappearance of these differences suggests that the initially observed higher infection rates in Yi patients may be related to external factors like their unique environmental exposures (e.g., biomass fuel use) and delayed healthcare-seeking behavior, rather than inherent biological differences between ethnic groups. This finding has important clinical implications: when evaluating and managing AECOPD patients, more attention should be paid to modifiable environmental and behavioral factors (such as improving indoor air quality and promoting smoking cessation) rather than overemphasizing ethnic differences. Of course, future studies still need to further clarify the nature of CT-detected pulmonary lesions through microbiological testing to better guide clinical decision-making.

Comorbidities are highly prevalent in COPD patients, which may have a significant impact on prognosis. Common contributing factors include advanced age, physical inactivity, poor diet, smoking, hypoxia, and systemic inflammation (42–44). Our findings reveal a complex interplay between COPD and comorbid conditions that exhibits distinct ethnic patterning. The significantly higher burden of metabolic and cardiovascular comorbidities (including coronary heart disease, hypertension, diabetes, and atrial fibrillation) observed in Han patients persists after rigorous PSM, suggesting these differences transcend traditional risk factors. Earlier observational reports have described that metabolic syndrome was significantly associated with an increased risk of COPD and the progression of COPD, and COPD can also predispose to metabolic syndrome (45, 46). Our research confirmed the relationship between COPD and metabolic syndrome. Four key mechanistic pathways may explain the observed ethnic disparities: (1) genetic variations in inflammatory and metabolic regulation, (2) distinct dietary patterns between Han and Yi populations, (3) differential healthcare utilization patterns, and (4) unique environmental co-exposures beyond smoking. The observed 10-year age difference between unmatched groups may reflect earlier disease onset in Yi populations due to heavier smoking exposure and biomass fuel use, warranting further investigation into ethnic variations in COPD pathogenesis. In addition, notable disparities were observed in acute-phase management between ethnic groups, with Han patients demonstrating significantly higher DBP, greater utilization of non-invasive ventilation, and increased ICU admission rates compared to Yi patients. This pattern may reflect either: (1) greater disease severity in Han patients as evidenced by more pronounced physiological derangements, or (2) systemic healthcare disparities affecting Yi populations, including limited access to medical resources, suboptimal treatment adherence, and potential health literacy gaps. These competing hypotheses warrant further investigation through controlled studies incorporating objective severity markers and healthcare accessibility metrics. Therefore, their awareness of disease treatment should be improved, treatment encouraged, and accurate intervention treatment programs should be developed for Yi patients. The overall in-hospital mortality rate (8%) exceeded previous Chinese reports (31). Notably, the lower mortality among Yi patients primarily reflects a methodological consideration: critically ill Yi patients frequently opted for discharge against medical advice (DAMA) due to cultural/religious preferences regarding end-of-life care (47). This systematic early discharge pattern, typically occurring during clinical deterioration, artificially reduced documented in-hospital mortality for this group. PSM analysis confirmed no statistically significant difference in formal in-hospital mortality rates between Yi and Han patients. However, the clinical trajectory of DAMA patients—typically discharged while hemodynamically unstable-strongly suggests these individuals would have experienced poor short-term outcomes if followed post-discharge (48). Thus, while the matched analysis shows comparable hospital mortality, this likely represents under-ascertainment of true mortality in the Yi population rather than equivalent disease outcomes. These findings highlight the critical importance of accounting for cultural factors in healthcare utilization patterns when interpreting ethnic disparities in respiratory outcomes.

Regarding laboratory findings, our observed CRP/PCT elevations (CRP > 10 mg/L; PCT ≥ 0.5 ng/ml) in both groups support the growing emphasis on inflammatory monitoring in AECOPD management, though interestingly without showing ethnic variation in these markers. The observed variations in biochemical parameters may reflect underlying ethnic differences in metabolic pathways, immune responses, and inflammatory regulation. Further investigation is warranted to elucidate the specific mechanisms driving these interethnic variations.

As for hospitalization costs, in the unmatched analysis, Yi patients demonstrated significantly lower total hospitalization costs compared to Han patients, and after PSM for key clinical variables, no significant differences in hospitalization costs. Potential explanations include: (1) the Yi population’s younger age and distinct comorbidity profile requiring less intensive interventions; (2) variations in healthcare utilization patterns; and (3) socioeconomic factors influencing treatment choices in this developing region. While these initial cost differences might indicate more cost-effective care for Yi patients, they could also suggest potential under-treatment, highlighting the complex interplay between clinical, socioeconomic, and ethnic factors in COPD management that warrants further investigation with more comprehensive socioeconomic data.

In this study, before matching, Yi patients were younger than Han patients, and the hospitalization cost was lower than for Han patients. To eliminate the influence of age on the study results, we conducted a matching analysis on age and found that the ventilation and ICU treatment of the Han patients were still higher than that of the Yi patients, which may be related to the more comorbidities and higher severity of diseases in the Han patients. The cost of COPD is closely related to disease severity; hospitalization cost is an important manifestation of the patient’s direct economic burden (49, 50). Similar to our results, studies have shown that the medical costs of COPD patients are a huge economic burden, and the older the COPD patients are, the greater the direct economic burden, and the higher the healthcare utilization and mortality (51–53). However, the significant differences in mortality should be noted with caution. Considering the specific ethnic habits of the Yi people, the Yi people often do not want to die in hospitals and have unique burial methods, and their in-hospital mortality does not reflect their real mortality (47), so there is no significant difference in mortality between the two groups after matching analysis.

Overall, our findings suggest several important considerations for clinical practice: standardized treatment protocols should be implemented to ensure equitable access to advanced respiratory support across ethnic groups; targeted smoking cessation and household air quality improvement programs are needed for Yi communities; cultural factors must be considered when interpreting outcomes in minority populations; population-specific reference ranges may be valuable for certain laboratory parameters (e.g., hemoglobin and PCO_2_). Future multicenter studies with comprehensive socioeconomic data and longitudinal follow-up are needed to further elucidate these ethnic variations and optimize care strategies.

Our study has several limitations that should be considered. First, the single-center design may introduce selection bias, as the included participants might not fully represent the broader Yi and Han populations in China. Regional healthcare-seeking behaviors and local diagnostic practices could further influence the generalizability of our findings. Second, missing data—particularly on lung function, height, and BMI—limited our ability to compare these key variables between the Yi and Han groups, potentially affecting the robustness of our conclusions. Third, while the medical record data were objectively extracted, certain patient-reported measures (e.g., smoking status, prior exacerbation history, and admission symptoms) are susceptible to recall or reporting bias. Additionally, unmeasured confounding factors (e.g., socioeconomic status or environmental exposures) could contribute to selection bias, as these were not systematically adjusted for in our analysis. Future multicenter studies with standardized data collection are needed to mitigate these limitations.

## Conclusion

In summary, this study reveals substantial ethnic disparities in the clinical manifestations, laboratory parameters, and therapeutic outcomes of AECOPD between Yi and Han populations. These disparities likely reflect complex interactions between genetic predisposition, environmental exposures (particularly biomass fuel use), and healthcare utilization patterns. The findings underscore the importance of developing ethnicity-specific clinical guidelines and public health interventions for COPD management in multi-ethnic regions of China. Future research should investigate the underlying mechanisms of these differences while addressing healthcare disparities through targeted community health programs for minority populations.

## Data Availability

The original contributions presented in this study are included in this article, further inquiries can be directed to the corresponding authors.

## References

[1] World Health Organization. *Chronic Obstructive Pulmonary Disease (COPD).* (2024). Available online at: https://www.who.int/news-room/fact-sheets/detail/chronic-obstructive-pulmonary-disease-(copd) (accessed November 6, 2024)

[2] Mu H Zhang Q. The application of diaphragm ultrasound in chronic obstructive pulmonary disease: a narrative review. *COPD.* (2024) 21:2331202. 10.1080/15412555.2024.2331202 38634575

[3] Christenson S Smith B Bafadhel M Putcha N. Chronic obstructive pulmonary disease. *Lancet.* (2022) 399:2227–42. 10.1016/S0140-6736(22)00470-6 35533707

[4] Wang C Xu J Yang L Xu Y Zhang X Bai C Prevalence and risk factors of chronic obstructive pulmonary disease in China (the China Pulmonary Health [CPH] study): a national cross-sectional study. *Lancet.* (2018) 391:1706–17. 10.1016/S0140-6736(18)30841-9 29650248

[5] World Health Organization. *The Top 10 Causes of Death.* (2024). (Available online at: https://www.who.int/news-room/fact-sheets/detail/the-top-10-causes-of-death (accessed August 7, 2024)

[6] Wang C Leung J Sin DD. A tale as old as time - the importance of accelerated lung aging in chronic obstructive pulmonary disease. *Expert Rev Respir Med.* (2025) 19:597–608. 10.1080/17476348.2025.2492800 40222750

[7] Chen T Hsu C Chang C Huang C Wang Y. Hyperbaric oxygen therapy attenuates carbon monoxide-induced lung injury by restoring mitochondrial dynamics and suppressing Pink1/Parkin-mediated mitophagy. *Environ Pollut.* (2025) 380:126521. 10.1016/j.envpol.2025.126521 40441279

[8] Ross B Doiron D Benedetti A Aaron S Chapman K Hernandez P Short-term air pollution exposure and exacerbation events in mild to moderate COPD: a case-crossover study within the CanCOLD cohort. *Thorax.* (2023) 78:974–82. 10.1136/thorax-2022-219619 37147124

[9] Ito K Barnes PJ. COPD as a disease of accelerated lung aging. *Chest.* (2009) 135:173–80. 10.1378/chest.08-1419 19136405

[10] Celli B Fabbri L Aaron S Agusti A Brook R Criner G An updated definition and severity classification of chronic obstructive pulmonary disease exacerbations: the rome proposal. *Am J Respir Crit Care Med.* (2021) 204:1251–8. 10.1164/rccm.202108-1819PP 34570991

[11] Maddocks M Kon S Singh S Man W. Rehabilitation following hospitalization in patients with COPD: can it reduce readmissions? *Respirology.* (2015) 20:395–404. 10.1111/resp.12454 25529496

[12] Mathioudakis A Janssens W Sivapalan P Singanayagam A Dransfield M Jensen J Acute exacerbations of chronic obstructive pulmonary disease: in search of diagnostic biomarkers and treatable traits. *Thorax.* (2020) 75:520–7. 10.1136/thoraxjnl-2019-214484 32217784 PMC7279206

[13] Prediletto I Giancotti G Nava S. COPD Exacerbation: why It is important to avoid ICU admission. *J Clin Med.* (2023) 12:3369. 10.3390/jcm12103369 37240474 PMC10218914

[14] Iheanacho I Zhang S King D Rizzo M Ismaila A. Economic burden of chronic obstructive pulmonary disease (COPD): a systematic literature review. *Int J Chron Obstruct Pulmon Dis.* (2020) 15:439–60. 10.2147/COPD.S234942 32161455 PMC7049777

[15] Gayle A Dickinson S Morris K Poole C Mathioudakis A Vestbo J. What is the impact of GOLD 2017 recommendations in primary care? - a descriptive study of patient classifications, treatment burden and costs. *Int J Chron Obstruct Pulmon Dis.* (2018) 13:3485–92. 10.2147/COPD.S173664 30498338 PMC6207393

[16] Gutiérrez Villegas C Paz-Zulueta M Herrero-Montes M Parás-Bravo P Madrazo Pérez M. Cost analysis of chronic obstructive pulmonary disease (COPD): a systematic review. *Health Econ Rev.* (2021) 11:31. 10.1186/s13561-021-00329-9 34403023 PMC8369716

[17] Qin J Wang G Liao Y Shang W Han D. High flow nasal therapy versus noninvasive ventilation for AECOPD with acute hypercapnic respiratory failure: a meta-analysis of randomized controlled trials. *Ann Intensive Care.* (2025) 15:64. 10.1186/s13613-025-01480-w 40360910 PMC12075079

[18] Hillas G Perlikos F Tzanakis N. Acute exacerbation of COPD: is it the stroke of the lungs? *Int J Chron Obstruct Pulmon Dis.* (2016) 11:1579–86. 10.2147/COPD.S106160 27471380 PMC4948693

[19] Fang L Gao P Bao H Tang X Wang B Feng Y Chronic obstructive pulmonary disease in China: a nationwide prevalence study. *Lancet Respir Med.* (2018) 6:421–30. 10.1016/S2213-2600(18)30103-6 29650407 PMC7185405

[20] Cai L Wang X Liu L Zhao Y Golden A. Socioeconomic differentials of trends in the prevalence and economic burden of chronic obstructive pulmonary disease in rural southwest China. *BMC Public Health.* (2023) 23:141. 10.1186/s12889-023-15096-x 36670366 PMC9854011

[21] Global Initiative for Chronic Obstructive Lung Disease. *Global Strategy for Prevention, Diagnosis and Management of COPD: 2025 Report.* (2025). Available online at: https://goldcopd.org/2025-gold-report/ (accessed May, 2025).

[22] Kirkpatrick DP Dransfield MT. Racial and sex differences in chronic obstructive pulmonary disease susceptibility, diagnosis, and treatment. *Curr Opin Pulm Med.* (2009) 15:100–4. 10.1097/MCP.0b013e3283232825 19532023

[23] Zarrabian B Mirsaeidi M. A trend analysis of chronic obstructive pulmonary disease mortality in the united states by race and sex. *Ann Am Thorac Soc.* (2021) 18:1138–46. 10.1513/AnnalsATS.202007-822OC 33347376

[24] Krishnan J Rajan M Banerjee S Mallya S Han M Mannino D Race and sex differences in mortality in individuals with chronic obstructive pulmonary disease. *Ann Am Thorac Soc.* (2022) 19:1661–8. 10.1513/AnnalsATS.202112-1346OC 35657680 PMC9528745

[25] McQuaid E Landier W. Cultural issues in medication adherence: disparities and directions. *J Gen Intern Med.* (2018) 33:200–6. 10.1007/s11606-017-4199-3 29204971 PMC5789102

[26] Mamary A Stewart J Kinney G Hokanson J Shenoy K Dransfield M Race and gender disparities are evident in COPD underdiagnoses across all severities of measured airflow obstruction. *Chronic Obstr Pulm Dis.* (2018) 5:177–84. 10.15326/jcopdf.5.3.2017.0145 30584581 PMC6296789

[27] Dezhi C Meili L Yingjian H Yiping H Yu T Weibo L. Population genetics of 27 Y-STRs for the Yi population from liangshan Yi autonomous prefecture, China. *Int J Legal Med.* (2021) 135:441–2. 10.1007/s00414-020-02249-5 32025783

[28] Yang Z Wang Y. Estimation of CDM emission reductions and benefit analysis of biomass stove introduction in Yi households. *Huanjing Kexue yu Jishu.* (2014) 37:201–4.

[29] Peng X. Comparative analysis of smoking status between Yi and Han male students. *J Xichang Coll Nat Sci.* (2014) 28:96–8.

[30] Li L Zhao N Ma X Sun F He B Qin Z Personalized variable vs fixed-dose systemic corticosteroid therapy in hospitalized patients with acute exacerbations of COPD: a prospective, multicenter, randomized, open-label clinical trial. *Chest.* (2021) 160:1660–9. 10.1016/j.chest.2021.05.024 34023318

[31] Zhang J Yi Q Zhou C Luo Y Wei H Ge H Characteristics, treatments, in-hospital and long-term outcomes among inpatients with acute exacerbation of chronic obstructive pulmonary disease in China: sex differences in a large cohort study. *BMC Pulm Med.* (2024) 24:125. 10.1186/s12890-024-02948-4 38468263 PMC10929097

[32] Berkius J Nolin T Mårdh C Karlström G Walther S. Characteristics and long-term outcome of acute exacerbations in chronic obstructive pulmonary disease: an analysis of cases in the Swedish intensive care registry during 2002-2006. *Acta Anaesthesiol Scand.* (2008) 52:759–65. 10.1111/j.1399-6576.2008.01632.x 18582304

[33] Montagnani A Mathieu G Pomero F Bertù L Manfellotto D Campanini M Hospitalization and mortality for acute exacerbation of chronic obstructive pulmonary disease (COPD): an Italian population-based study. *Eur Rev Med Pharmacol Sci.* (2020) 24:6899–907. 10.26355/eurrev_202006_21681 32633383

[34] Salvi S Barnes P. Chronic obstructive pulmonary disease in non-smokers. *Lancet.* (2009) 374:733–43. 10.1016/S0140-6736(09)61303-9 19716966

[35] Thawanaphong S Nair P. Contemporary concise review 2024: chronic obstructive pulmonary disease. *Respirology.* (2025) 30:574–86. 10.1111/resp.70062 40437348 PMC12231764

[36] Pandey A Verma A Singh A Kant S Chaudhary S Bajpai J The severity of non-smoking chronic obstructive pulmonary disease is correlated with biomass fuel exposure and COPD assessment test score. *Lung India.* (2024) 41:251–8. 10.4103/lungindia.lungindia_304_23 38953187 PMC11302787

[37] Chan K Kurmi O Bennett D Yang L Chen Y Tan Y Solid fuel use and risks of respiratory diseases. A cohort study of 280,000 Chinese never-smokers. *Am J Respir Crit Care Med.* (2019) 199:352–61. 10.1164/rccm.201803-0432OC 30235936 PMC6363974

[38] Song X Cong S Fan J Wang N Wang W Fang L. [Prevalence and influencing factors of severe respiratory infections in childhood among residents of China, 2019-2020]. *Zhonghua Liu Xing Bing Xue Za Zhi.* (2024) 45:1617–25. 10.3760/cma.j.cn112338-20241009-00620 39681417

[39] Allinson J Chaturvedi N Wong A Shah I Donaldson G Wedzicha J Early childhood lower respiratory tract infection and premature adult death from respiratory disease in Great Britain: a national birth cohort study. *Lancet.* (2023) 401:1183–93. 10.1016/S0140-6736(23)00131-9 36898396

[40] Ding Y Xu J Yao J Chen Y He P Ouyang Y The analyses of risk factors for COPD in the Li ethnic group in Hainan, People’s Republic of China. *Int J Chron Obstruct Pulmon Dis.* (2015) 10:2593–600. 10.2147/COPD.S86402 26664107 PMC4670019

[41] Wang Y Wang J Lu Z Zhou Q Cao Y Du Y Global, regional, and national burden of lower respiratory infections and chronic obstructive pulmonary disease, 1990-2021: a systematic analysis from the global burden of disease study 2021. *Infection.* (2025): 10.1007/s15010-025-02566-0 Online ahead of print. 40455385

[42] Recio Iglesias J Díez-Manglano J López García F Díaz Peromingo J Almagro P Varela Aguilar J. Management of the COPD patient with comorbidities: an experts recommendation document. *Int J Chron Obstruct Pulmon Dis.* (2020) 15:1015–37. 10.2147/COPD.S242009 32440113 PMC7217705

[43] Shen L Lv J Li J Zhou J Wang X. Managing osteoporosis in COPD. *Endocr Metab Immune Disord Drug Targets.* (2024) 24:896–901. 10.2174/1871530323666230913105752 37711118

[44] Fabbri L Celli B Agustí A Criner G Dransfield M Divo M COPD and multimorbidity: recognising and addressing a syndemic occurrence. *Lancet Respir Med.* (2023) 11:1020–34. 10.1016/S2213-2600(23)00261-8 37696283

[45] Li S Zhang T Yang H Chang Q Zhao Y Chen L Metabolic syndrome, genetic susceptibility, and risk of chronic obstructive pulmonary disease: the UK Biobank Study. *Diabetes Obes Metab.* (2024) 26:482–94. 10.1111/dom.15334 37846527

[46] Xie Q Xu S Wan Q Tong N. Metabolic syndrome, small airway dysfunction and the mediating role of inflammation. *Sci Rep.* (2025) 15:12555. 10.1038/s41598-025-97326-3 40221581 PMC11993575

[47] Luo C. The implied view of life and death in Yi ethnic funeral customs. *Yanhuang Dili.* (2021):44–9.

[48] Tan S Feng J Joyce C Fisher J Mostaghimi A. Association of hospital discharge against medical advice with readmission and in-hospital mortality. *JAMA Netw Open.* (2020) 3:e206009. 10.1001/jamanetworkopen.2020.6009 32525546 PMC7290410

[49] Benmaamar S Es-Sabbahi B Taghyioullah Haiba M Omari M El Harch I Youbi M Economic burden of chronic obstructive pulmonary disease in Morocco: a cost of illness study. *Monaldi Arch Chest Dis.* (2025) 95: 10.4081/monaldi.2024.2745 [Online ahead of print]. 38226692

[50] Gan L He X Wu J. Impact of moderate and severe exacerbations on clinical prognosis and economic burden of chronic obstructive pulmonary disease in China. *Expert Rev Pharmacoecon Outcomes Res.* (2025): 10.1080/14737167.2025.2507425 Online ahead of print. 40382707

[51] Zhu B Wang Y Ming J Chen W Zhang L. Disease burden of COPD in China: a systematic review. *Int J Chron Obstruct Pulmon Dis.* (2018) 13:1353–64. 10.2147/COPD.S161555 29731623 PMC5927339

[52] Fang X Wang X Bai C. COPD in China: the burden and importance of proper management. *Chest.* (2011) 139:920–9. 10.1378/chest.10-1393 21467059 PMC7125604

[53] Jehloh L Songwathana P Kitrungrote L Bourbonnais A. Perspectives of family caregivers and nurses on hospital discharge transitional care for Muslim older adults living with COPD: a qualitative study. *BMC Nurs.* (2024) 23:273. 38659051 10.1186/s12912-024-01943-8 PMC11044287

